# Multiple Cellular Responses to Serotonin Contribute to Epithelial Homeostasis

**DOI:** 10.1371/journal.pone.0017028

**Published:** 2011-02-24

**Authors:** Vaibhav P. Pai, Nelson D. Horseman

**Affiliations:** Department of Molecular and Cellular Physiology, University of Cincinnati, Cincinnati, Ohio, United States of America; University of Birmingham, United Kingdom

## Abstract

Epithelial homeostasis incorporates the paradoxical concept of internal change (epithelial turnover) enabling the maintenance of anatomical status quo. Epithelial cell differentiation and cell loss (cell shedding and apoptosis) form important components of epithelial turnover. Although the mechanisms of cell loss are being uncovered the crucial triggers that modulate epithelial turnover through regulation of cell loss remain undetermined. Serotonin is emerging as a common autocrine-paracine regulator in epithelia of multiple organs, including the breast. Here we address whether serotonin affects epithelial turnover. Specifically, serotonin's roles in regulating cell shedding, apoptosis and barrier function of the epithelium. Using *in vivo* studies in mouse and a robust model of differentiated human mammary duct epithelium (MCF10A), we show that serotonin induces mammary epithelial cell shedding and disrupts tight junctions in a reversible manner. However, upon sustained exposure, serotonin induces apoptosis in the replenishing cell population, causing irreversible changes to the epithelial membrane. The staggered nature of these events induced by serotonin slowly shifts the balance in the epithelium from reversible to irreversible. These finding have very important implications towards our ability to control epithelial regeneration and thus address pathologies of aberrant epithelial turnover, which range from degenerative disorders (*e.g.;* pancreatitis and thyrioditis) to proliferative disorders (*e.g.;* mastitis, ductal ectasia, cholangiopathies and epithelial cancers).

## Introduction

Epithelial tissue constitutes tightly bound layers of cells that form the surface of body (epidermis, cornea) and define the internal linings of organs, glands and ducts (digestive tract, mammary gland, bile duct, etc. [1]. Polarized epithelial tissues provide a] barriers against external environments and compartmentalization of internal organs, and b] regulation of glandular secretions and movement of fluid and ions across these compartments. Epithelial tissue homeostasis is the maintenance of such a functional polarized cell-layer. This epithelial tissue homeostasis is a function of balances between cell production, differentiation and loss of spent cells (cell turnover). Appropriate regulation of the rate of epithelial turnover is important, as there are epithelia with high turnover rates (*e.g.;* gut -4-5 days– [1]–[3]) and those with a low turnover rate (lung – 6months [4]). Alternatively, regulated periods of imbalances between turnover events (proliferation, differentiation and loss) drive developmental and adaptive changes in the epithelial tissue architecture and function. Archetypes of cyclical changes in epithelial homeostasis are the proliferation, differentiation and regression in the epithelia of the mammary gland during the lactation cycle, and the uterus during a menstrual cycle [5]–[7].

Cell shedding and apoptosis are two important components of the cell turnover process. Cell shedding is observed in a wide range of highly proliferative epithelial tissues including, but not limited to, retina and intestine [1], [5], [7]. It is believed to be involved in removal of spent differentiated epithelial cells; however its trigger signal, mechanism and function remain largely unstudied [8]–[11]. The importance of apoptosis as a primary means of cell loss during epithelial turnover is well documented, especially its role in developmental processes and adult tissue homeostasis [5], [12], [13]. Both epithelial cell shedding and apoptosis by elimination of defective cells from the epithelium may create a deterrent against transformation [14]. A coordinated interplay between endocrine and autocrine-paracrine factors regulates epithelial turnover (proliferation, shedding and apoptosis) [7]. Appropriate regulation of epithelial loss is critical, as aberrant turnover is responsible for numerous pathologies ranging from those due to impaired cell loss (mastitis, ectasia, cholangiopathies, cholestasis,pancreatic blockade - [14]) to those due to exacerbated cell loss (pancreatitis and thyroiditis [14]–[16]). In addition, aberrant turnover is hallmark of cancer progression [17]–[19].

Serotonin (5-hydroxytryptophan, 5-HT) is evolutionarily among the most conserved molecules [20]. Apart from its classically studied role as a neurotransmitter it has multiple functions in the periphery, ranging from maintaining vascular tone to regulating bone metabolism [21], [22]. Analysis of recent literature reveals an emerging role of 5-HT as an autocrine-paracrine regulator of epithelium in various organ systems including gut, prostate, pancreas, lung, liver and salivery glands [23]–[29]. Our previous studies identified mammary gland to be an epithelium that synthesizes and secretes 5-HT, and that serotonin regulates mammary gland involution in an autocrine-paracrine fashion [30].

Mammary gland involution is induced by the absence of suckling stimulus, resulting in accumulation of milk within the gland (milk stasis). Involution occurs in two phases; reversible and irreversible [31], [32]. The reversible phase is characterized by inhibition of epithelial milk production, cell shedding, and apoptosis. However, returning the suckling stimulus takes the gland back into lactation. The irreversible phase is induced by breakdown of epithelial junctions and large scale tissue remodeling consisting of epithelial regression. Serotonin is highly induced upon milk stasis and in turn inhibits epithelial milk protein synthesis [30] . Serotonin also holds the capacity to disrupt mammary epithelial tight junctions [33], [34].

In this report we try to understand 5-HT's role in regulating mammary epithelial turnover, especially during the reversible and irreversible phases. We show that 5-HT dynamically affects both cell shedding and apoptosis. Moreover, the apoptosis is induced in the replenishing cells. Because of the staggered (on time scale) manner of induction of these events, 5-HT is able to transition the epithelium from functioning to a regressing epithelium.

## Results

### 5-HT induces mammary epithelial cell shedding *in vivo*

Events associated with involution are regulated by both endocrine and local factors [31], [32] . In order to determine the influence of local factors like 5-HT on cell shedding we performed a teat sealing experiment. In a lactating mouse a subset of mammary glands are surgically sealed (to induce stasis and involution). Because of continued suckling on the unsealed teats the endocrine factors are maintained whereas the local factors are induced only in the sealed glands. The glands were harvested 36 hours after teat sealing and analyzed by routine histology and hematoxylin and eosin staining.

**Figure 1:**
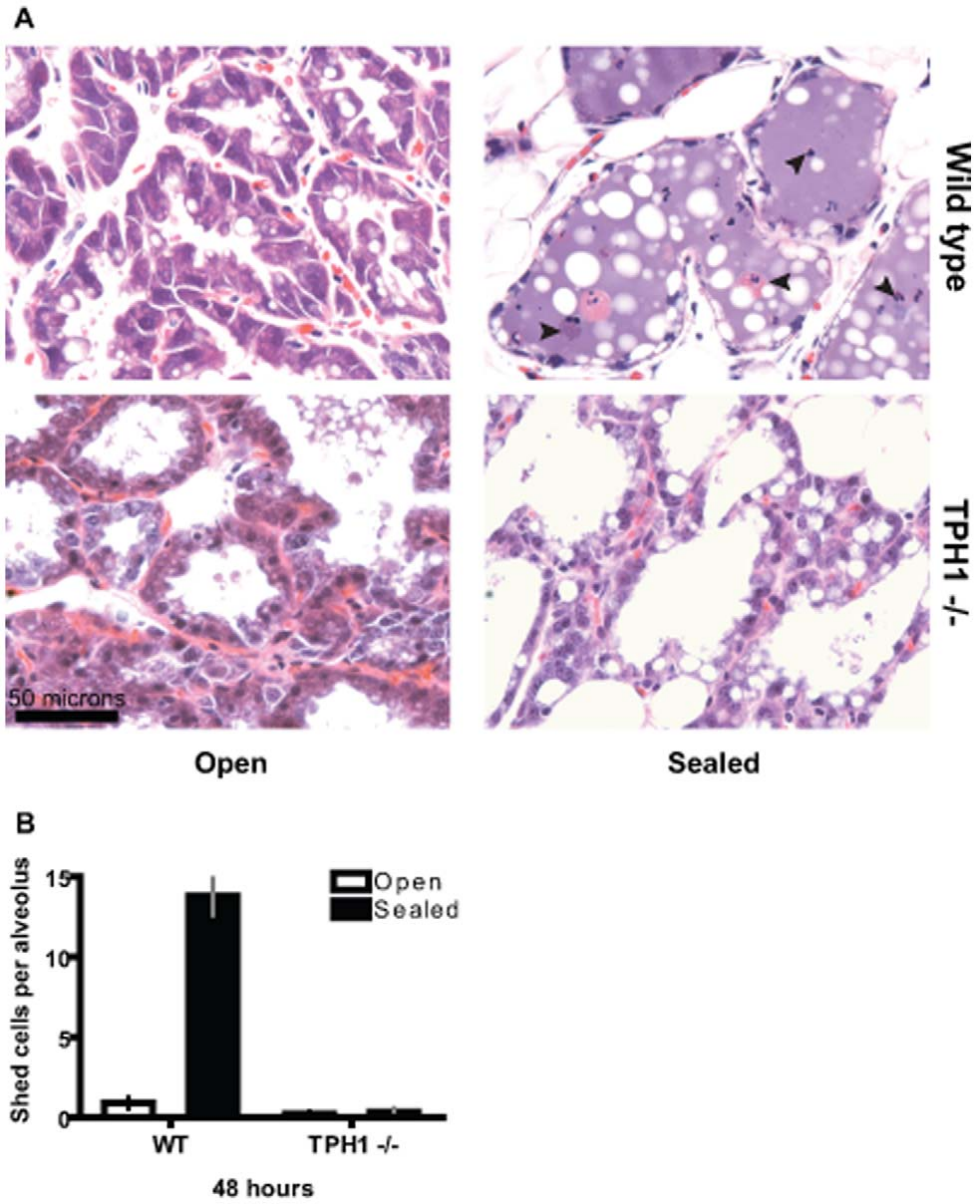
Local 5-HT regulates milk stasis– induced epithelial cell shedding. (A) Contralateral mammary gland number 4 from lactating wild type and TPH1 −/− mice after teat-sealing. The glands were harvested 48 h after teat-sealing. Wild type sealed glands show large number of shed cells (most undergoing anoikis) in the alveoli (black arrowheads). Shed cells were rare in sealed TPH1 −/− glands. (B) Quantification of numbers of cells shed into the lumen per alveolus from 5 different alveoli each from 5 independent glands. Data are represented as mean +/− S.E.M.

In the context of lactation, the mammary epithelium was highly active and columnar in appearance (Figure 1A WT open). Minimal to undetectable amounts of cell shedding, and no noticeable apoptosis was observed in the epithelium during lactation (Figure1A WT open and Figure 1B). In the involuting glands (sealed) there was a dramatic increase in the epithelial cells being shed into the lumen (Fig. 1A WT sealed black arrowheads and Figure 1B). Since, only local factors are brought into play at this point, it suggests that cell shedding is induced by local factors. In order to address whether 5-HT affects cell shedding we performed teat-sealing in TPH1 knockout mice. Sealed TPH1−/− mice failed to show the characteristic increase in shed cells (Figure 1A TPH−/− sealed and Figure 1B). Thus, absence of 5-HT is associated with absence of cells shed into the lumen. This suggested that 5-HT was involved in regulating the cell shedding seen in response to milk stasis and involution. Note that all the glands depicted were still in the reversible phase of involution [31], [32].

### Replenishment of the mammary epithelium by multipotent cells

**Figure 2:**
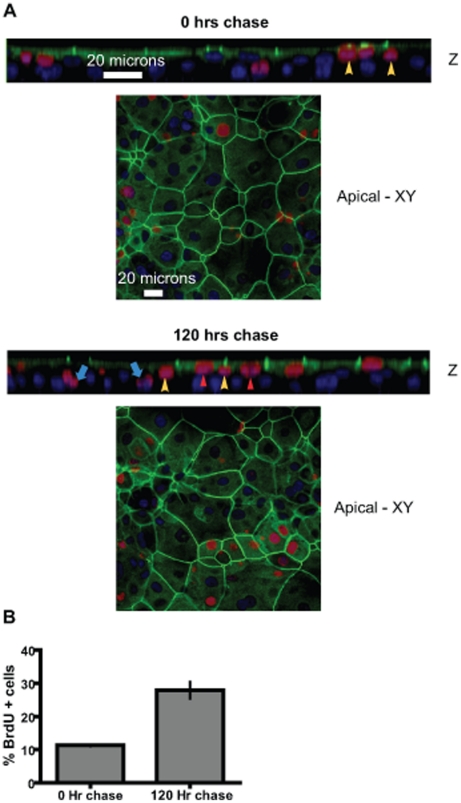
Suprabasal multipotent cells give rise to both luminal and basal cells. A BrdU pulse-chase experiment in MCF10A Transwell® cultures show the location of dividing epithelial cells. The MCF10A transwell® cultures were labeled with BrdU for 24 hours followed by medium change and continued cultivation of the cultures for the designated periods of time. (A) Representative confocal Z-section and apical XY-sections immunofluorescently stained for BrdU (red), ZO1 (green) and nuclei (blue). Note the suprabasal location of BrdU + cells (yellow arrowheads) at 0 h of chase. After 120 hours chase the BrdU label is seen in both the luminal cells (ZO1+) (red arrowheads) and the basal cells (blue arrows). Suprabasal putative multipotent cells found wedged between the luminal and basal layers are also observed (yellow arrowheads). (B) Quantification of BrdU + cells after 0 hours chase and 120 hours chase indicates a 2.5 fold increase in BrdU + cells. The data is expressed as means +/− S.E.M.

Epithelial cell replenishment is an important component of epithelial homeostasis. In order to understand this phenomenon and the influence of 5-HT on it, we have established an *in vitro* model of MCF10A human mammary duct epithelium cultured on permeable membranes [35]. Like *in vivo* epithelium, these MCF10A cultures consists of mucin1 (MUC1)+, barrier forming, Zona Occludens 1 (ZO1)+ luminal cells, and an underlying CD10+ layer of basal cells [35]. By cloning stem cells under non-adherent condtions we have shown that these cultures contain multipotent cells able of giving rise to both basal and luminal lineages. Here we have used a bromodeoxyuridine (BrdU) pulse-chase experiment to visualize the putative multipotent cells and confirm the dynamic process that gives rise to basal and luminal cells. A BrdU pulse for 24 hours labeled a small population (∼8%) of cells that were situated in a suprabasal location (Figure 2 and Figure S1-0 hrs – yellow arrowheads). Chasing the BrdU label for 120 hours showed the label being transferred to both the barrier forming ZO1+ luminal cells (Figure 2 and Figure S1 – 120 hrs – red arrowheads) and the underlying basal cells (blue arrows). By day 5, 25% cells were BrdU–labeled (Figure 2B). This indicates that BrdU–labeled suprabasal cells divide and generate both the luminal and basal layer cells. Interestingly, chasing the BrdU signal for 9 days resulted in dramatic dilution of the label in most cells (Figure S2 – red arrowheads). However, a small fraction of cells (∼6%), situated suprabasally, retained the bright BrdU label for at least 9 days (Figure S2 – yellow arrowheads).

### 5-HT induces mammary epithelial cell shedding

**Figure 3:**
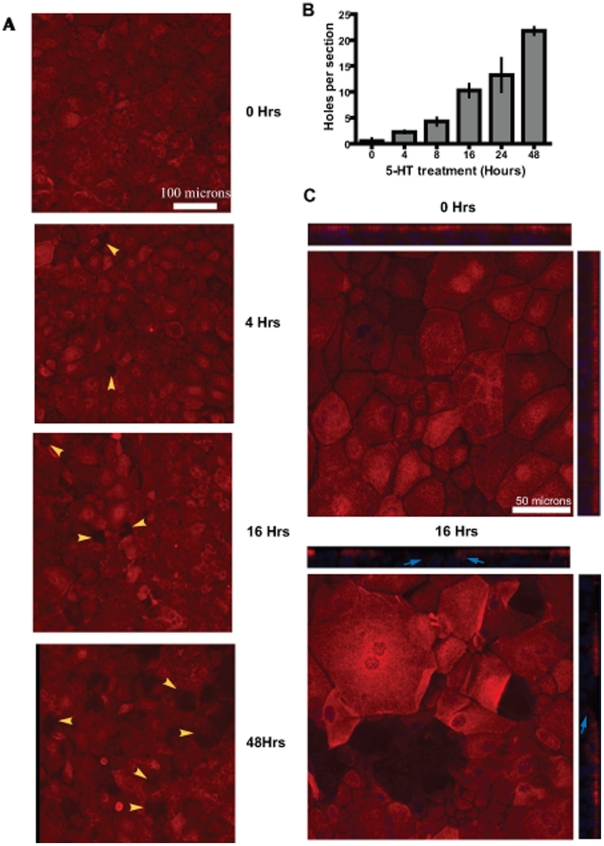
5-HT induces mammary epithelial cell shedding. Confocal images of cultures treated with 5-HT (10^−3^ M) for indicated time periods showing a time–dependent induction of epithelial cell shedding by 5-HT. (A) The cultures were stained for a luminal marker (MUC1) (red) and nuclei (blue). Note the holes in the epithelium seen as areas lacking MUC1 staining (yellow arrowheads). (B) Quantification of holes in the epithelium seen upon 5-HT treatment. At least 4 different sections for each time point were analyzed. Counting was discontinued after 48 hour due to lack of resolution with respect to holes created by single cells and multiple adjacent cells. The number of holes appears to progressively increase with time of exposure to 5-HT. The data is expressed as means +/− S.E.M. (C) Shows magnified representative XY and Z sections of cultures at 0 hrs and 16 hrs after exposure to 5-HT. At 0 hrs the epithelial lining is straight and intact. By 16 Hrs the shed cells have left behind an undulating epithelial lining. The cells surrounding/lining the holes from underneath, express low to normal levels of MUC1 (blue arrows).

**Figure 4:**
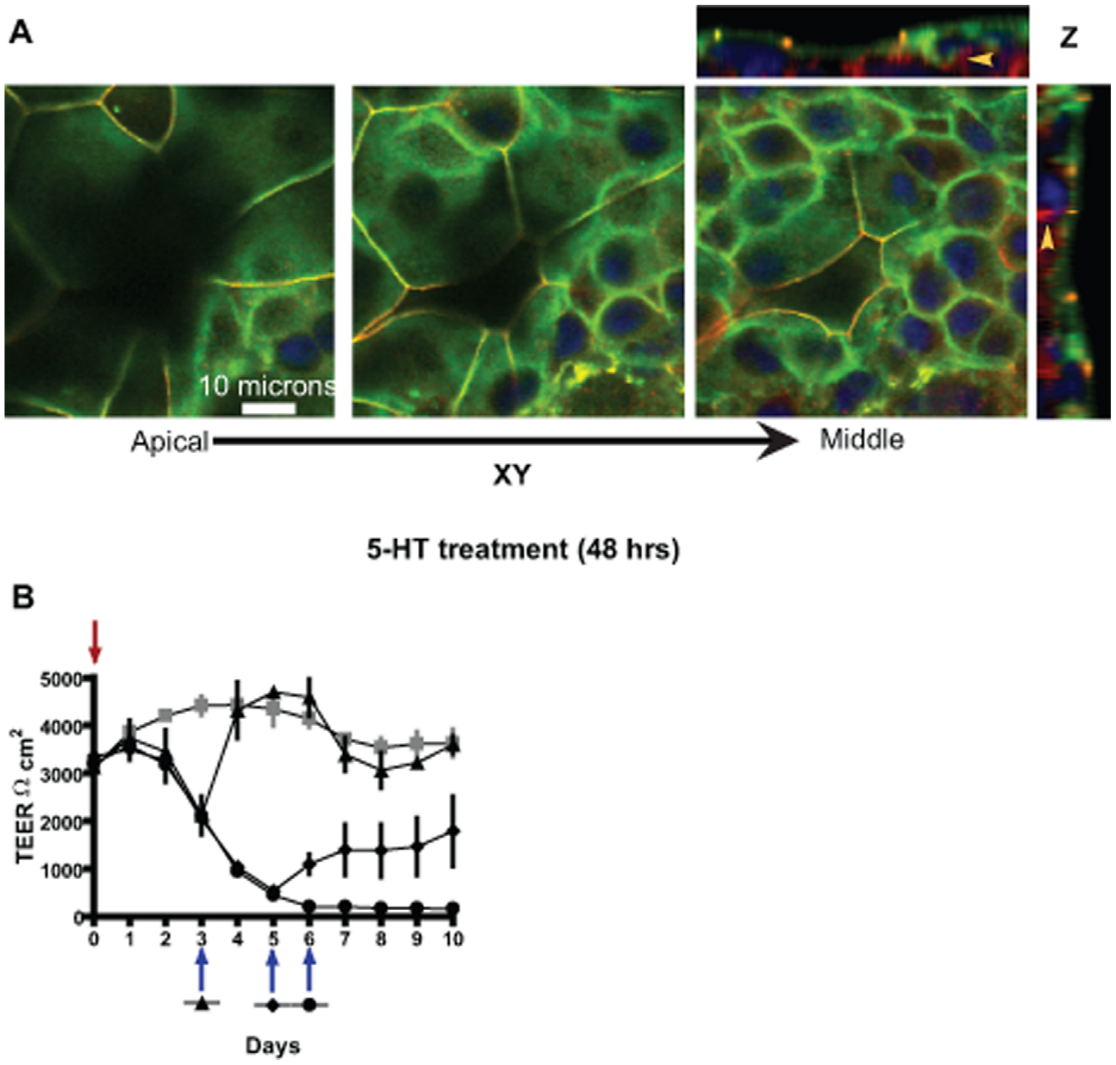
5-HT–mediated disruption of epithelial tight junctions is reversible in a time dependent manner. (A) Confocal images of MCF10A Transwell® cultures treated with 5-HT for 48 hours. Representative serial XY sections and Z-section are depicted. The cultures were stained for tight junction proteins occludin (green), ZO1 (red) and nuclei (blue). The serial sections show that the holes left behind by shed cells are lined underneath by differentiated cells, forming tight junctions with their neighbors. The underlying cells also show increased ZO1 and occludin (yellow arrowheads). (B) Trans-epithelial electrical resistance (TEER - Ωcm^2^) measured on cultures left untreated (line with grey squares) or treated with 5-HT (line with filled triangle, circle and diamond). Red arrow indicates the time-point when 5-HT was added whereas blue arrow indicate the time-points when 5-HT was withdrawn for a particular experimental group. Early withdrawal of 5-HT leads to a full recovery. However, prolonged exposure to 5-HT cause a loss of TEER recovery capacity of the epithelium.

In order to determine 5-HT's effect on cell shedding we treated cultures with 5-HT for various time periods and then visualized the luminal layer using MUC1 stain. Holes in the MUC1+ epithelium were generated by the shedding of cells (Figure 3). In untreated cultures shedding was relatively rare, with 1–2 holes per 4–5 sections (Figure 3A and B). However, as early as 4 hours after 5-HT treatment we observed frequent holes in the epithelium (Figure 3A yellow arrowheads). The number of holes increased with the time of exposure to 5-HT (Figure 3B). In the magnified Z-section image at 16 hours after 5-HT treatment (Figure 3Cand S3) the undulating nature of the epithelium left behind by the shed cells was obvious. In addition, at the site of holes left behind by the shed cells, the underlying cells began to show MUC1 expression (Figure 3C Arrows). This observation suggests a replacement of the shed cells from the suprabasal cell layer. This conclusion is further supported by the observation that although increased cell shedding was observed in response to 5-HT beginning 4 hours, the barrier functions as measured by transepithelial electrical resistance (TEER), is not substantially compromised until 48 hours (Figure 4).

### Cell shedding occurs without compromising barrier function

To address how 5-HT induced cell shedding without compromising barrier function, we looked for tight junctions (ZO1 and Occludin localization) within the holes left behind by the shed cells after 5-HT treatment (Figure 4A). As seen in figure 4A and Movie S1, the cells adjacent to the holes form tight junctions with the cells lining the hole from underneath (Arrows). Also an increase in the amount of tight junctional proteins (ZO1 and Occludin) is observed in the cells directly underneath the luminal cell layer. These observations indicate that as a luminal cell is induced to shed (in response to 5-HT), the adjacent cells in the luminal layer and the underlying layer cooperate to maintain the barrier function.

### Reversibility of 5-HT action on mammary epithelium

Our previous studies have shown that 5-HT influences mammary epithelial tight junctions in a biphasic manner resulting in a delayed tight junction disruption. Since mammary gland involution consists of a reversible phase we looked at the reversible nature of 5-HT action on the epithelium. We treated the transwell® cultures with 5-HT and removed 5-HT from the cultures at different time-points with respect to changes in TEER (measure of tight junctions). Withdrawal of 5-HT after a 33% drop in TEER resulted in complete restoration to control (untreated) TEER levels (Figure 4B). Cell shedding halted and the epithelium looked similar to the control epithelium (Figure S4). Withdrawal of 5-HT after an 80% drop in TEER resulted in only partial recovery of the barrier function and the TEER, although high, did not return to the control levels (Figure 4B). Finally, 5-HT withdrawal after a 93% drop in TEER resulted in no recovery of the barrier function. At this point the multilayered epithelium lost its composition. Many cells were lost, and the remaining cells formed a quasi-monolayer of cells without tight junctions (Figure S4). These observations suggest that the time of exposure of the epithelium to 5-HT affects its regenerative capacity, presumably through the effect of 5-HT on the multipotent suprabasal cells. This results in a gradual decline in the ability to restore a functional epithelium until a point of no return is reached.

### 5-HT induces delayed apoptosis in mammary epithelium

**Figure 5:**
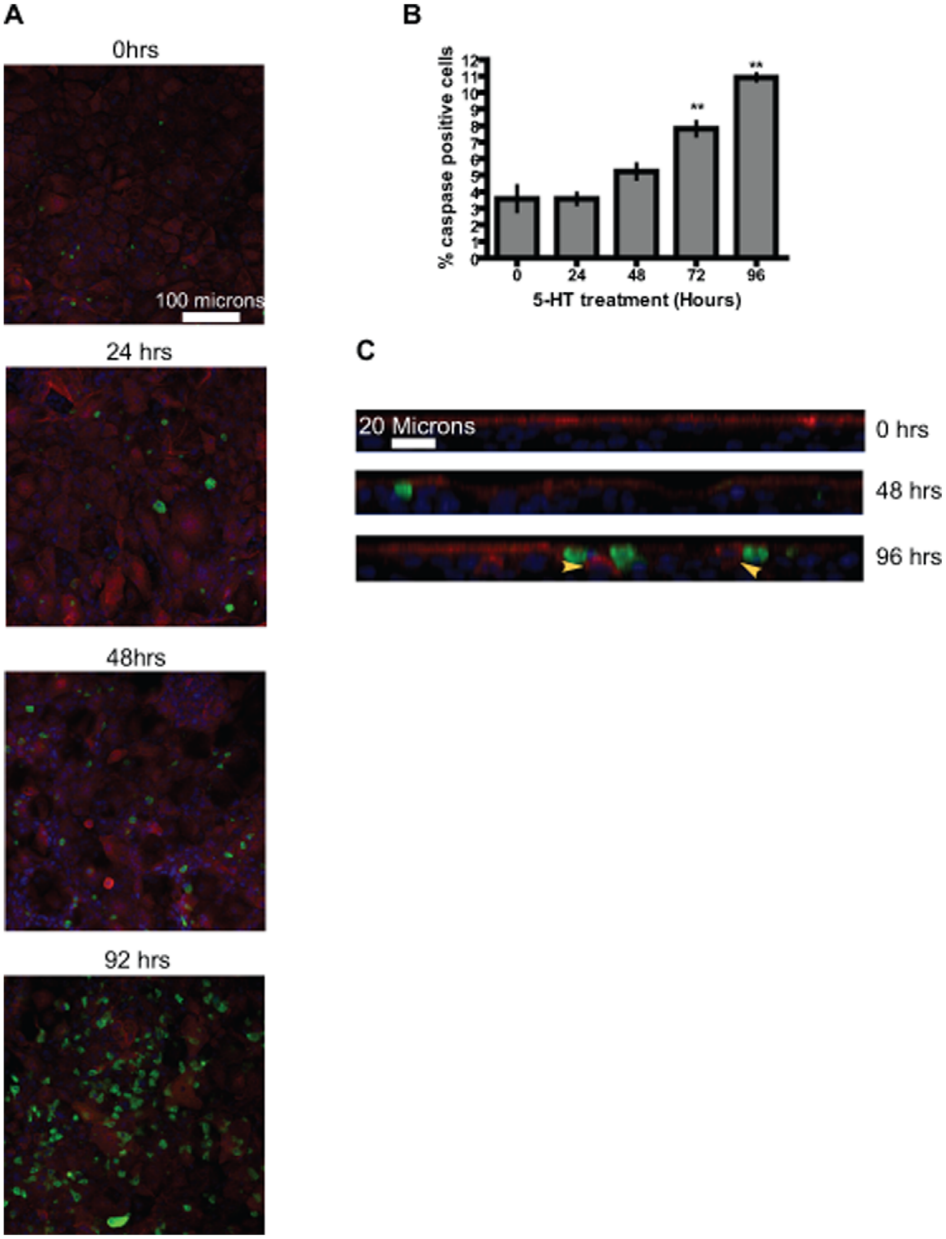
5-HT induces delayed intra-epithelial apoptosis in MCF10A Transwell® cultures. (A) Representative confocal XY images of cultures after 5-HT treatment for the indicated time periods showing 5-HT–induced apoptosis. The sections were stained for MUC1 (red), and apoptosis (cleaved caspase 3, green). Nuclei were stained blue. (B) Quantification of cells staining positive for cleaved caspase 3. Cells from at least four independent sections were counted for each time-point. The data are expressed as mean +/− S.E.M. **p<0.01 (One way Anova). Significant induction of apoptosis is not seen until 72 hrs of 5-HT treatment. (C) Representative confocal Z-section images of cultures after 5-HT treatment for indicated time-points. The sections were stained for luminal epithelial marker (MUC1) (red) and apoptosis marker (cleaved caspase 3) (green). Nuclei were stained blue. Note that the caspase positive cells are located mainly in the suprabasal cell layer. Also certain cells from the suprabasal layer stain positive for MUC1 (yellow arrowheads).

Since apoptosis is an important contributor to epithelial turnover, one way 5-HT could affect the epithelium is through induction of apoptosis. To understand 5-HT action on apoptosis we treated the cultures with 5-HT and stained for cleaved caspase3 (apoptosis marker). As seen in figure 5A, 5-HT induced apoptosis in the epithelium, but this effect of 5-HT was significantly delayed, with the increase in apoptosis not observed until 72 hours (Figure 5B). Notice the highly undulating membrane (due to cell shedding) as observed in the Z-sections of the cultures (Figure 5C). The apoptosis induced by 5-HT was mainly observed in the suprabasal layer of cells, which are responsible for replenishing the lost luminal cells (Figure 5C). Very few apoptotic cells were observed in the basal cells, which largely remained refractory to the apoptotic effect of 5-HT (Figure S4). Certain cells from the suprabasal layer which were not yet undergoing apoptosis expressed MUC1. However, this may not be enough to keep up with the increased cell loss. Thus, 5-HT, first through induction of luminal cell shedding and then through induction of apoptosis, shifts the balance towards cell loss, resulting in regression of the epithelium.

## Discussion

Epithelia perform very crucial functions of compartmentalization, especially within glandular and secretory tissues. Our studies address the influence of 5-HT on epithelial homeostasis. The mammary epithelium secretes and responds to 5-HT in ways that promote local secretory homeostasis [30]. Independent studies have shown secretion of 5-HT by epithelia of other ductal organs, *viz;* prostate, pancreas, lung, gut, salivary gland [23]–[29]. In order to further study the roles of 5-HT in epithelial homeostasis we developed an *in vitro* model of human mammary ductal epithelium using MCF10A, a non-transformed human mammary epithelial cell line [35].

### 5-HT induces mammary epithelial cell shedding

During lactation mammary epithelium bears the enormous burden of synthesizing and secreting milk for the nourishment of the young one. Hence, the actively lactating mammary epithelium is very dynamic, differentiated and columnar in structure, as illustrated in Fig. 1. Consistent with previous studies [36] we observed low epithelial turnover rates during lactation. After a period of milk stasis there is a dramatic increase in epithelial cells shedding, with detached cells undergoing anoikis [37]. Unlike the normal glands, there was an absence of shed cells after teat sealing in TPH1-knockout mammary glands. This indicated that 5-HT is required for normal cell turnover associated with involution of the glands. After milk stasis there is a dramatic change in the shape of the epithelial cells (squamous transition), which appears to induce changes in cellular Ca2+ homeostasis [38]. The resulting calcium overload is an important trigger for apoptosis [38]. Interestingly, 5-HT3A receptor is expressed in mammary epithelium [39]. 5-HT3A is a ligand-gated cation channel, and may play an important role in Ca2+ homeostasis and thus cellular apoptosis. The failure of TPH1 knockout mammary glands to undergo squamous transition after milk stasis shows that 5-HT is essential for normal morphological changes in response to distension of the mammary glands.

### Barrier function is maintained during 5-HT–induced cell shedding

As the epithelial cell is being shed, it becomes rounded, and leaves behind a depression (hole) in the epithelium. MUC1 is newly expressed in the cells underlying the shed cells, suggesting that the suprabasal cell layer contains progenitor cells that can be driven to differentiate into luminal cells, replacing the shed cells and maintaining the barrier. This hypothesis is supported by the observation that these underlying cells have also newly express tight junction proteins. Such localized differentiation of progenitor cells could be through an indirect action of 5-HT, but is more like that the juxtacrine and paracrine signaling from the luminal cells drive their differentiation. The details of analogous signaling mechanisms have been worked out in some detail in the gut epithelium where enterocytes are shed and replaced at a high rate [40], [41]. Whether the mammary epithelium shares some of those mechanisms is, as yet, unknown.

In the mammary epithelium 5-HT induces both cell shedding and disruption of tight junctions (current studies and [33], [34]). While these two processes are coordinated, and both are driven by 5-HT, they occur on a very different time scales in this model system, indicating that they depend on different mechanisms. Under the conditions in these experiments, cell shedding begins by 4 hours of treatment, whereas significant disruption of tight junctions does not occur until after 48 hours. Cell shedding independent of a compromise in barrier function has also been reported in MDCK kidney cells and intestinal epithelium [8]–[11].

### Mammary epithelium is replenished by multipotent cells

The mammary ductal epithelium is composed of 3 cell layers; a] luminal cells, b] basal cells (including myoepithelium) and c] suprabasal cells that may comprise a stem/progenitor niche [1], [6], [42], [43]. Through clonogenic assays we have previously shown the presence of mulipotent cells in MCF10A cultures [35]. Here, using BrdU pulse-chase and label retention assays we have been able to visualize the position of these cells, and demonstrate their ability to give rise to the other epithelial cell layers. The observation that by day 5, 25% of the population was BrdU–labeled suggests a significant turnover of the epithelium. However, whether this is truly representative of human mammary epithelium or a characteristic of the culture system remains to be determined. The long-term (>9 days) retention of bright BrdU labeling by a subset of suprabasal cells suggests that these cells may undergo asymmetric cell division with strand retention as observed in mouse mammary epithelium [44]. Another possibility is that most of the turnover observed over the period of 10 days is due to only 1–2% of the labeled cells, whereas the others remain quiescent throughout this period [43].

### 5-HT–induced apoptosis within the epithelial membrane influences its replenishing capacity

Withdrawal of 5-HT after a partial % drop in TEER resulted in a rapid recovery of the epithelium (within 24 hours). Hence, it can be inferred that the epithelium is able to rapidly replace the shed cells, and reverse the tight junction disruption initiated by 5-HT. However, larger scale opening of the tight junctions resulted in irreversible changes to the epithelium. This appears to be the result of a change in the replenishing capacity of the epithelium, thus resulting in inability of the epithelium to restore itself. This idea is supported by the induction of apoptosis within the suprabasal cell layer, which appears to contain the stem and progenitor cell niches. We suggest that the luminal progenitors, which normally replenish the membrane, are lost upon extended exposure to 5-HT, resulting in a loss of the regenerative capacity of the epithelium. Presumably, the hormonal changes that occur during pregnancy are able to repopulate the luminal progenitor niche, and allow for the re-growth of a fully functional epithelial membrane.

The sequential time course of events induced by 5-HT, shedding by 4 hours, disruption of tight junctions by 48 hours, and apoptosis by 72 hours, implies that these events could be regulated independently by 5-HT. The human mammary epithelium expresses four 5-HT receptors *viz;* 5-HT_1D_, 5-HT_2B_, 5-HT_3A_ and 5-HT_7_ [39], which could mediate separate events. We have been successful in attributing the tight junction regulation to the 5-HT_7_ receptor and it's downstream signaling [33], [34]. However, we have not yet attributed functions to the other receptors, and this remains an active area of study. It is also likely that indirect effects of 5-HT, through induction of secondary mediators such as cytokines could ultimately be responsible for controlling later events. This is supported by our observation that 5-HT induces expression of cytokines within the mammary epithelium (data not shown). These two possibilities (direct and indirect 5-HT action) are not mutually exclusive. Changes in cell-cell interactions, caused by shedding and tight junction disruption, could provide important intracellular signals that induce secondary mediators.

In conclusion we have been able to show here that 5-HT regulates epithelial turnover though three specific events: cell shedding, tight junction breakdown, and intra-epithelial apoptosis. Two of these events (shedding and tight junctions disruption) are reversible, whereas the intra-epithelial apoptosis is associated with irreversible changes to epithelium. These findings are potentially significant for other tissues, as 5-HT is a common factor in regulation of various ductal epithelia, and thus may provide a signal for fine tuning the regenerative capacity of these epithelia. This might play crucial therapeutic roles in treatment of pathologies ranging from cholangiopathies to epithelial tumors.

## Methods

### Ethics statement

All experiments were conducted under protocols that were approved (# 05-01-11-01) by the Institutional Animal Care and Use Committee at University of Cincinnati.

### Mice

TPH1 −/− and corresponding controls (TPH1+/+) were on a C57BL/6J inbred genetic background. Female mice (Wild-type and TPH1 −/−) underwent surgery at peak lactation (day 10) under general anesthesia to seal the nipples on only one side of the body. The dams were then returned to their pups to continue nursing.

### Teat-sealing experiment

Mice upon reaching peak lactation (day 10) were anesthetized. Nipples of abdominal glands 3, 4 and 5 from only one side of the body were sealed using a suture and surgical glue. The glands on the other side of the body were left unsealed. The dams were returned back to their pups. Visual confirmation of pups resuming suckling was obtained. 36 hours after teat-sealing the mice were sacrificed and the glands were harvested.

### Histology

Tissues were fixed in 4% paraformaldehyde, embedded in paraffin, sectioned at 5 µm, and stained with conventional hematoxylin-eosin reagents (Integrative Morphology Core, Cincinnati Children's Hospital and Medical Center).

### Cell Culture

An immortalized human mammary epithelial cell line, MCF10A (ATCC, Manassass, VA **-** CRL-10317™), was used. The normal growth medium for MCF10A was DMEM:F12 (1∶1; Cellgro) with 2 mM glutamine, containing 5% horse serum, insulin (10 µg ml–1; Gibco), hydrocortisone (0.5 µg ml–1; Sigma), epithelial growth factor (EGF; 20 ng ml–1; Upstate), 1 cIU ml–1 penicillin, 0.1 µg ml– 1 streptomycin, 0.25 µg ml– 1 amphotericin B (Cellgro). Cells were grown in monolayer to 90%–95% confluency, trypsinized, and counted for seeding onto permeable supports (Transwell, Corning; 0.4 µm pores, polyester) in normal growth medium. MCF10A cells were seeded on 12-well Transwells at 1.25×10^5^ cells/cm2. Both chambers of media were changed strictly on a 24-h schedule, unless otherwise noted. Where indicated, serotonin (5-hydroxytryptamine creatine sulfate; Sigma) was administered in both chambers. Transepithelial electrical resistance (TEER) was measured daily with an Epithelial Volt-Ohm Meter (EVOM; World Precision Instruments), prior to media change.

### Immunofluorescence and imaging

Cells were grown on permeable supports to peak TEER (∼3000Ωcm^2^), fixed by a brief incubation in 4% paraformaldehyde (in phosphate-buffered saline), and immunostained as floating sections of membranes. The floating sections were permeabilized in 0.1% Triton X-100 and incubated in borate buffer pH 8.5 (80 mM boric acid, 20 mM sodium borate) overnight at 75°C for antigen retrieval. The following antibodies and fluorescent stains were used: rabbit polyclonal anti-occludin (5 µg ml–1; Zymed), and mouse anti-mucin 1 (0.8 µg ml–1; Santa Cruz). TO-PRO-3 iodide dye (1 µM; 642/661; Molecular Probes) was used to visualize nuclei. Images were collected by using a Zeiss LSM510 Confocal Microscope. The excitation sources were argon lasers at 488 nm and 543 nm and a helium/neon laser at 633 nm, with emission collection at 505–530 nm, 560–615 nm, and 650–720 nm, respectively.

## Supporting Information

Figure S1 **Suprabasal location of multipotent cells and their ability to give rise to both luminal and basal cells:** A BrdU pulse chase experiment where theMCF10A Transwell® cultures were labeled with BrdU for 24 hours followed by continued cultivation of the cultures for the mentioned period of time. Representative confocal XY-serial sections (Apical, Middle and Bottom) of cultures immunofluorescently stained for BrdU (red), ZO1 (green) and nuclei (blue), indicating the suprabasal location of BrdU + cells within the cultures. After 120 hours (5 days) chase the BrdU label is seen in both the luminal cells and the basal cells, as well as suprabasal cells.
(TIF)

Figure S2 **Intense BrdU label retention by a subpopulation of suprabasal cells:** A BrdU pulse chase experiment where the MCF10A Transwell® cultures were labeled with BrdU for 24 hours, followed by continued cultivation of the cultures for 216 hrs. (A) Representative confocal XY and Z section of cultures immunofluorescently stained for BrdU (red), ZO1 (green) and nuclei (blue). The yellow arrowheads mark the intense BrdU label-retaining cells, which are located in the suprabasal position. Cells weakly BrdU + in the luminal cells (ZO1+) and basal cells are marked with red arrowheads. (B) Quantification of only intensely BrdU + cells from 4 independent sections after 216 hours chase. The data are represented as mean +/− S.E.M.
(TIF)

Figure S3 **Shed cells leave a depression/hole in the epithelium, creating an undulating epithelial lining:** Confocal Z-section image of cells immunostained for MUC1 (red) and nuclei (blue), showing cell in the process of shedding (yellow arrowhead). Note the rounding of the cell being shed and the hole caused by its departure.
(TIF)

Figure S4 **Irreversible action of 5-HT on the epithelium:** Representative confocal Z-sections of control (untreated) and 5-HT–treated MCF10A Transwell® cultures. The cultures were stained for tight junction proteins occludin (green) and ZO1 (red). The nuclei were stained blue. Control section shows 2–3 cell layers with punctate tight junctions in the luminal cell layer. 5-HT treated cultures show a quasi-monolayer of cells, deficient in tight junctions.
(TIF)

Movie S1 **5-HT induced cell shedding is associated with underlying cells forming tight junctions with the neighboring cells:** confocal Z-sections series of MCF10A transwell® cultures treated with 5-HT for 48 hours. The cultures were stained for tight junction proteins occludin (green), ZO1 (red) and nuclei (blue).
(AVI)
