# Supplementary figures and images for: Multiple Cellular Responses to Serotonin Contribute to Epithelial Homeostasis
Source: PLoS One. 2011 Feb 24;6(2):e17028. doi: 10.1371/journal.pone.0017028 (PMC3044750; doi:10.1371/journal.pone.0017028)

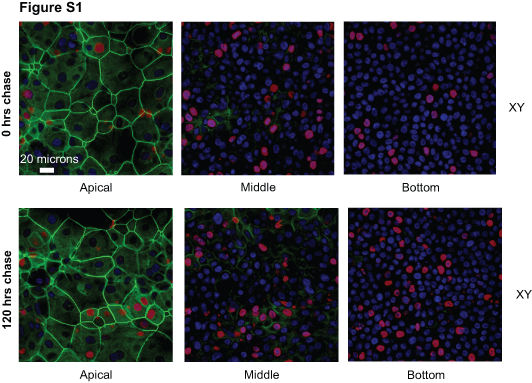

Supplement: Figure S1 — Suprabasal location of multipotent cells and their ability to give rise to both luminal and basal cells: A BrdU pulse chase experiment where theMCF10A Transwell® cultures were labeled with BrdU for 24 hours followed by continued cultivation of the cultures for the mentioned period of time. Representative confocal XY-serial sections (Apical, Middle and Bottom) of cultures immunofluorescently stained for BrdU (red), ZO1 (green) and nuclei (blue), indicating the suprabasal location of BrdU + cells within the cultures. After 120 hours (5 days) chase the BrdU label is seen in both the luminal cells and the basal cells, as well as suprabasal cells. (TIF) [file pone.0017028.s001.tif]

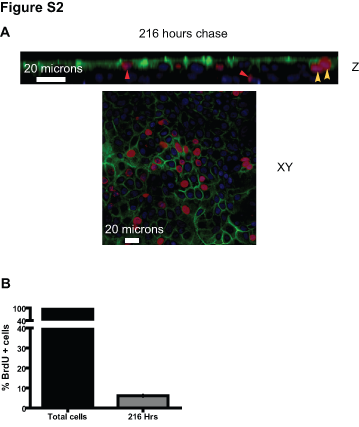

Supplement: Figure S2 — Intense BrdU label retention by a subpopulation of suprabasal cells: A BrdU pulse chase experiment where the MCF10A Transwell® cultures were labeled with BrdU for 24 hours, followed by continued cultivation of the cultures for 216 hrs. (A) Representative confocal XY and Z section of cultures immunofluorescently stained for BrdU (red), ZO1 (green) and nuclei (blue). The yellow arrowheads mark the intense BrdU label-retaining cells, which are located in the suprabasal position. Cells weakly BrdU + in the luminal cells (ZO1+) and basal cells are marked with red arrowheads. (B) Quantification of only intensely BrdU + cells from 4 independent sections after 216 hours chase. The data are represented as mean +/− S.E.M. (TIF) [file pone.0017028.s002.tif]

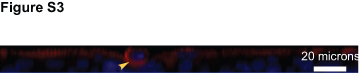

Supplement: Figure S3 — Shed cells leave a depression/hole in the epithelium, creating an undulating epithelial lining: Confocal Z-section image of cells immunostained for MUC1 (red) and nuclei (blue), showing cell in the process of shedding (yellow arrowhead). Note the rounding of the cell being shed and the hole caused by its departure. (TIF) [file pone.0017028.s003.tif]

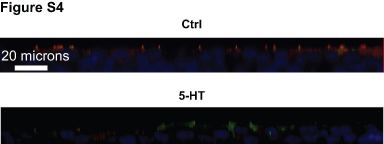

Supplement: Figure S4 — Irreversible action of 5-HT on the epithelium: Representative confocal Z-sections of control (untreated) and 5-HT–treated MCF10A Transwell® cultures. The cultures were stained for tight junction proteins occludin (green) and ZO1 (red). The nuclei were stained blue. Control section shows 2–3 cell layers with punctate tight junctions in the luminal cell layer. 5-HT treated cultures show a quasi-monolayer of cells, deficient in tight junctions. (TIF) [file pone.0017028.s004.tif]
